# Supplementary material for: MetaQTL: a package of new computational methods for the meta-analysis of QTL mapping experiments
Source: BMC Bioinformatics. 2007 Feb 8;8:49. doi: 10.1186/1471-2105-8-49 (PMC1808479; doi:10.1186/1471-2105-8-49)
Supplement: Additional File 7 — MetaQTL Package : jar file and tutorial. This Zip archive contains both the MetaQTL JAR file and the files of the tutorial. [file 1471-2105-8-49-S7.zip › org.inra.metaqtl/doc/org/metaqtl/bio/util/NumberFormat.html]

NumberFormat


|  |  |  |
| --- | --- | --- |
| **Overview** \| **Package** \| **Class** \| **Use** \| **Tree** \| **Deprecated** \| **Index** \| **Help** | |  |
| **PREV CLASS**   **NEXT CLASS** | **FRAMES**    **NO FRAMES**     **All Classes** |  |
| SUMMARY: NESTED \| FIELD \| CONSTR \| METHOD | DETAIL: FIELD \| CONSTR \| METHOD |  |


---


## org.metaqtl.bio.util Class NumberFormat

```
java.lang.Object
  org.metaqtl.bio.util.NumberFormat
```

---

``` public final class NumberFormat extends java.lang.Object ```

**Author:**
:   jveyrier
    TODO To change the template for this generated type comment go to
    Window - Preferences - Java - Code Style - Code Templates

---

**Constructor Summary**

`NumberFormat()`


| **Method Summary** | |
| --- | --- |
| `static java.lang.String` | `formatDouble(double d)` |
| `static double` | `parseDouble(java.lang.String value)`             This method parse the given string to a double value. |

**Methods inherited from class java.lang.Object**

`clone, equals, finalize, getClass, hashCode, notify, notifyAll, toString, wait, wait, wait`

**Constructor Detail**

### NumberFormat

```
public NumberFormat()
```


**Method Detail**

### formatDouble

```
public static java.lang.String formatDouble(double d)
```

:   **Parameters:**: `d` - **Returns:**

---


### parseDouble

```
public static double parseDouble(java.lang.String value)
```

:   This method parse the given string to a double value. If the
    string is not valid `Double.NaN` is returned.
    Note that this method is 'coma'/'dot' tolerant.

    :   **Parameters:**: `value` - **Returns:**


---


|  |  |  |
| --- | --- | --- |
| **Overview** \| **Package** \| **Class** \| **Use** \| **Tree** \| **Deprecated** \| **Index** \| **Help** | |  |
| **PREV CLASS**   **NEXT CLASS** | **FRAMES**    **NO FRAMES**     **All Classes** |  |
| SUMMARY: NESTED \| FIELD \| CONSTR \| METHOD | DETAIL: FIELD \| CONSTR \| METHOD |  |


---
